# Decoding the Role of Extracellular Polymeric Substances in Enhancing Nitrogen Removal from High-Ammonia and Low-C/N Wastewater in a Sequencing Batch Packed-Bed Biofilm Reactor

**DOI:** 10.3390/polym15061510

**Published:** 2023-03-17

**Authors:** Zheng Fan, Xin Zhou

**Affiliations:** 1 College of Environmental Science and Engineering, Taiyuan University of Technology, Taiyuan 030024, China; e0950181@u.nus.edu; 2 Department of Biological Sciences, National University of Singapore, Singapore 117543, Singapore

**Keywords:** extracellular polymeric substances, nitrogen removal, high-ammonia and low-C/N wastewater, polyurethane foam, sequencing batch packed-bed biofilm reactor

## Abstract

Although the role of extracellular polymeric substances (EPSs) as a viscous high-molecular polymer in biological wastewater treatment has been recognized, in-depth knowledge of how EPSs affect nitrogen removal remains limited in biofilm-based reactors. Herein, we explored EPS characteristics associated with nitrogen removal from high-ammonia (NH_4_^+^-N: 300 mg/L) and low carbon-to-nitrogen ratio (C/N: 2–3) wastewater in a sequencing batch packed-bed biofilm reactor (SBPBBR) under four different operating scenarios for a total of 112 cycles. Scanning electron microscopy (SEM), atomic force microscopy (AFM), and Fourier-transform infrared (FTIR) analysis revealed that the distinct physicochemical properties, interface microstructure, and chemical composition of the bio-carrier were conducive to biofilm formation and microbial immobilization and enrichment. Under the optimal conditions (C/N: 3, dissolved oxygen: 1.3 mg/L, and cycle time: 12 h), 88.9% ammonia removal efficiency (ARE) and 81.9% nitrogen removal efficiency (NRE) could be achieved in the SBPBBR. Based on visual and SEM observations of the bio-carriers, biofilm development, biomass concentration, and microbial morphology were closely linked with nitrogen removal performance. Moreover, FTIR and three-dimensional excitation–emission matrix (3D-EEM) spectroscopy demonstrated that tightly bound EPSs (TB-EPSs) play a more important role in maintaining the stability of the biofilm. Significant shifts in the number, intensity, and position of fluorescence peaks of EPSs determined different nitrogen removal. More importantly, the high presence of tryptophan proteins and humic acids might promote advanced nitrogen removal. These findings uncover intrinsic correlations between EPSs and nitrogen removal for better controlling and optimizing biofilm reactors.

## 1. Introduction

With the fast development of industrialization, the discharge of heavily polluted industrial wastewater has dramatically increased. Particularly, it is noteworthy that some high-strength ammonia wastewater with a low COD to total nitrogen (C/N), generated from coking, coal chemical, fertilizer, tannery, pharmaceutical, slaughter, landfill, livestock, and poultry breeding industries, has become a major challenge to efficient wastewater treatment [1]. Therefore, it is urged to explore novel technologies to solve the pollution of high-strength nitrogenous wastewater.

For a long time, activated sludge processes (ASPs) based on two-step nitrification and denitrification have been extensively applied for biological nitrogen removal (BNR) from wastewater in wastewater treatment plants (WWTPs) [2]. First, ammonia is oxidized to nitrite and then to nitrate via autotrophic bacteria in the nitrifying reactor. While in the denitrifying reactor, nitrate was reduced to nitrite and finally to nitrogen gas. For the application of high-ammonia wastewater treatment, however, the ASP-based BNR approaches have distinct limitations, such as low biomass concentration and short sludge retention time [3], resulting in poor adaptability of autotrophic nitrifying sludge and inhibited nitrogen removal in the presence of high free ammonia (FA) [4]. More importantly, the external addition of organic carbons is normally necessary to enhance the denitrification from wastewater with limited C/N ratios [5]. As such, process complexity and operating costs would be significantly increased for WWTPs. 

Alternatively, the biofilm attached to the filling materials consists of a more complex microecological structure of microbial cells and extracellular substances [6]. The biofilm process could have a robust ability to retain biomass and a powerful resistance to inhibitors. Particularly, the biofilm also provides a desirable growth environment for immobilizing slow-growing microbes (e.g., nitrifiers) [7] and more endogenesis organic carbons for heterotrophic denitrifiers owing to an extremely long mean cell retention time (MCRT). Correspondingly, improved nitrification and denitrification could be simultaneously achieved in a single-stage biofilm reactor, which offers a more robust and cost-effective BNR technology compared to two-stage ASPs [8]. The sequencing batch biofilm reactor (SBBR), combining the advantages of both the biofilm and batch operation, is one of the most popular biofilm reactors [9]. So far, a variety of SBBRs, including sequencing batch packed-bed biofilm reactor (SBPBBR) [10], sequencing batch moving-bed biofilm reactor (SBMBBR) [11], and sequencing batch membrane biofilm reactor (SBMBR) [12] have been developed and proven to be reliable for BNR from wastewater. Although these biofilm reactors have been widely applied for nitrogen removal from both domestic and industrial wastewater, an in-depth understanding of physicochemical property and characterization of the biofilm itself remains still limited, which become an obstacle for further optimizing nitrogen removal capacity in biofilm reactors from a microscale perspective.

Extracellular polymeric substances (EPSs) secreted by microbial cells are a complex mixture of high-molecular-weight polymers, mainly including proteins, polysaccharides, nucleic acids, lipids, and other polymeric compounds [13]. As a core of the biofilm, EPSs account for over 90% of the dry weight of the total biomass [14]. EPSs are produced by the adsorption and bio-conversion of organic matter in the wastewater and the hydrolysis of cell lysis during wastewater treatment. EPSs offer powerful resistance and resilience for the self-protection of microbial cells from harmful environmental conditions by forming water-filled reticular structures that bind the cells within the biofilm to each other [15,16]. Due to the shear force of water and airflow, as well as microorganisms’ metabolisms, the biofilms can be shed and renewed over time, resulting in dynamic changes in EPS properties. In particular, the EPS component contents can alter significantly depending on the external environment, such as wastewater characteristics and operating conditions. Such environmental variations have decisive impacts on treatment efficiency and stability of the biofilm process [17,18].

It was generally acknowledged that EPSs were mostly secreted by heterotrophic bacteria through the biotransformation of organic matter in aerobic biofilm reactors [19,20,21]. However, EPSs have also been confirmed to play a critical role, and their component and function variations have been closely associated with nitrogen removal performance in some autotrophic nitrification and anammox biofilm reactors [22,23,24,25], although the metabolism of these autotrophic microorganisms is slow. In a one-stage biofilm reactor, nitrification and denitrification often coexist in the presence of organics and ammonium [25]. Therefore, it is necessary to unravel the correlation between EPSs and nitrogen removal to instruct the optimization of biofilm systems. In previous research, the influence of changing individual environmental factors, including temperature [22], influent COD [23], C/N [24], dissolved oxygen (DO) [25], and microbial inhibitors [26] on EPSs in nitrogen removal of SBBR systems has been widely investigated. Nevertheless, a comprehensive understanding of EPSs in advancing nitrogen removal in SBBR is still lacking in the case of the combined effect of multiple parameters. Therefore, a further study focusing on the correlation between EPSs and nitrogen removal under simultaneous adjustments of multiple parameters is required.

In this study, an SBPBBR filled with polyurethane (PU) foam was used for high-ammonia and low-C/N wastewater treatment. To optimize nitrogen removal, C/N ratios, cycle time, and DO were chosen to be key adjusting parameters. The purposes were to (1) first explore the bio-carriers’ physio-chemical properties, the biofilm morphology, and the biological phase through scanning electron microscopy (SEM), atomic force microscopy (AFM), and Fourier-transform infrared (FTIR); (2) investigate long-term nitrogen removal performance under different operating conditions; and (3) reveal the link between EPS shifts and nitrogen removal capacity using FTIR and three-dimensional excitation–emission matrix (3D-EEM) fluorescence spectroscopy. This study helps to gain more insights into the role of EPSs in indicating nitrogen removal from high-strength wastewater using biofilm reactors.

## 2. Materials and Methods

### 2.1. Experimental Setup

**Figure 1:**
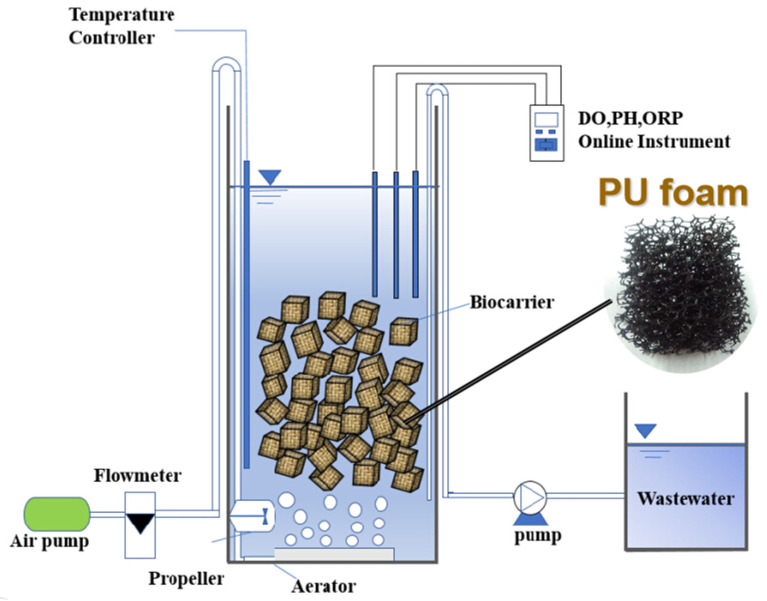
Schematic diagram of the SBPBBR system.

A lab-scale plexiglass-made SBPBBR system (Figure 1) was established in this experiment. The working volume was approximately 5 L with a 150 mm inner diameter and 350 mm height. The commercial PU foam purchased from Hangzhou Zijing Environmental Protection Engineering Co., Ltd. (Hangzhou, China) was used as a bio-carrier. The filling ratio was 45% (*v*/*v*). The PU carrier is cellular or expanded material made by a double explosion of oxygen and hydrogen, with a pore size of 2–7 mm and mutual penetration, ensuring microorganisms’ attachment in all directions of the whole filler. The parameters of bio-carriers are summarized in Table S1. The oxygen was provided by an aeration disk located at the bottom by an air compression pump. The aeration rate and reaction aeration time were controlled via a gas flow meter and time controller, respectively. The propeller fixed at the bottom of the side wall provided the mixing and stirring. The system was kept at room temperature. The reactor was operated at SBR mode, including 15 min filling, 23.5 h/11.5 h reaction, and 15 min decanting in a cycle of 24/12 h. Additionally, the volume ratio of the decanting and the total was 1/3.

### 2.2. Reactor Start-Up

The reactor was fed by high-strength synthetic wastewater composed of COD, nitrogen, and phosphorus sources provided by glucose, NH_4_Cl, and KH_2_PO_4_, respectively. The pH was approximately 7.6. Sodium bicarbonate was added to supplement the alkalinity required for nitrification. In addition, a small amount of nutrient solution was added to the feed to supplement trace elements for the growth of microorganisms. The composition and trace elements of synthetic wastewater are listed in Table 1. The activated sludge (mixed liquor suspended solids: 3100 mgMLSS/L) taken from the SBR process in a municipal wastewater treatment plant in Jingzhong city, Shanxi Province, China, was used as the inoculum. The sludge was first washed with deionized water thrice to remove impurities fully. After washing the sludge, 1.5 L inoculated sludge and 3.5 L synthetic wastewater were pumped together into the reactor and then stood for 24 h. A small amount of aeration was carried out for 8–10 h to develop the young biofilm. After 14–16 h precipitation, 0.5 L of the supernatant was decanted and replaced by fresh wastewater. After five-day repetitive operations, the influent flow rate gradually increased, and the aeration intensity was enhanced to ensure the rapid hanging of the biofilm. The successful start-up of the SBPBBR reactor was completed after 60 cycles.

### 2.3. Experiment Operation

After the acclimatization and start-up, the reactor was operated in the steady state. To investigate the combined impact of different operating conditions, i.e., C/N ratio, cycle time (CT), and DO, on the EPS in nitrogen removal, four consecutive operational scenarios (R1–R4) were set up in Table 2. The total experiment lasted for 122 cycles.

### 2.4. Chemical Analysis

COD, ammonia nitrogen, nitrate nitrogen, and nitrite nitrogen were determined using the reagent rapid determination methods (HACH DR1900, Loveland, CO, USA). The total nitrogen concentration was regarded as the sum of all nitrogen species. Dissolved oxygen (DO), pH, and oxidation-reduction potential (ORP) were monitored by a multiparameter (WTW3420, Munich, Germany). The fixed biomass concentration of the bio-carrier was calculated as VSS (g/L) based on the dry weight method [27].

### 2.5. SEM Observation

Scanning electron microscopy (SEM) was used to observe the surface morphology of biofilms and microbial phases under four different scenarios. The biofilm samples were rinsed with 0.1 mol/L phosphate buffer (pH 7.4) for 5 min and then hardened in glutaraldehyde (2.5% concentration) for 12 h; rinsed again with phosphate buffer twice for 10 min each; dehydrated in ethanol solutions with concentrations of 30%, 50%, 70%, 80%, 90%, 95%, and 100%, respectively. Finally, the samples were immersed in isoamyl acetate solution at 50%, 70%, 80%, 90%, 95%, and 100%, respectively, for 15 min. After the freeze-drying and coating, the treated samples were observed by scanning electron microscope (JSM-7001F, Tokyo, Japan). Microbial community was analyzed by Illumina Miseq high-throughput sequencing performed by Sangon Botech Co., Ltd. (Shanghai, China).

### 2.6. AFM Spectroscopy

The original PU foam was observed by atomic force microscopy (Park nx10, Suwon, Republic of Korea) at a non-contact scanning mode with an SPM probe made of silicon with an elasticity factor of 10–130 N/m, a maximum scanning range of 50 μm × 50 μm, and a resonance frequency of 204–497 kHz. XEI software provided by AFM was used to analyze the surface morphology characteristics of the PU foam.

### 2.7. Extraction of EPSs

In this experiment, a piece of PU foam during the stabilization period of each scenario was taken out into a beaker, distilled water was added and shaken for 10 min in an oscillator at 150 r/min to dislodge the biofilm from the bio-carrier in distilled water, and after settling for several minutes, the supernatant was removed; thus, the biofilm was obtained. The EPSs were extracted after the centrifugation tube by the following steps [28]. Generally, EPSs include tightly bound EPSs (TB-EPSs), loosely bound EPSs (LB-EPSs), and soluble EPSs (S-EPSs) [29]. The obtained biofilm was directly centrifuged and frozen at 2000× *g* for 15 min, and the supernatant was taken and filtered through a 0.45 μm microfiltration membrane to obtain S-EPSs. Then, the buffer was added to the centrifuge tube to replenish the original volume, centrifuged at 5000× *g* for 15 min, and the supernatant was taken and filtered through a 0.45 μm microfiltration membrane to obtain LB-EPSs, where the pH of the buffer was 7.0, and it contained 1.3 mmol/L Na_3_PO_4_, 2.7 mmol/L NaH_2_PO_4_, 6 mmol/L NaCl, and 0.7 mmol/L KCl. Continue to add a buffer to the remaining sludge in the previous step to the original volume, water bath at 60 °C for 30 min, freeze centrifuge at a centrifugal force of 5000× *g* for 20 min, and then filter through a 0.45 μm membrane. The supernatant was filtered, and the TB-EPSs were obtained [30].

### 2.8. Spectrum Analysis of EPSs

The FTIR analysis was performed using an infrared spectrometer (Vertex 70, Bruker, Bremen, Germany) with wave number range from 400 to 4000 cm^−1^, with a detection resolution of 4 cm^−1^. The obtained EPSs were placed in a Petri dish and frozen overnight at −20 °C in a refrigerator, then continuously freeze-dried in a freeze-dryer for about 12 h. 10 mL of water samples were firstly dried at 40 °C. The solid sample was powdered and mixed with dry KBr (1:100 wt ratio) and then pressed into a pellet under 10 tons of pressure for 1 min. Additionally, a blank sample was used to establish the spectral baseline for IR spectroscopy.

The 3D-EEM spectrum analysis was recorded using a 3D fluorescence spectrometer (Varian, Palo Alto, CA, USA) with the scanning excitation wavelength from 200 to 550 nm and emission wavelengths from 200 to 550 nm. The excitation and emission slit widths were set to 10 nm, and the scanning speed was maintained at 1200 nm/min with a scanning interval of 10 nm. The spectra of deionized water were used as blank samples.

All the obtained spectrum data were plotted and analyzed using Origin 2017 (OriginLab, Northampton, MA, USA).

## 3. Results and Discussion

### 3.1. Property of Original Bio-Carriers

As the characteristics of bio-carriers as the core for wastewater purification in the biofilm process could affect microbial enrichment and biofilm development, thus affecting nitrogen removal performance in SBBR [31], it was necessary to analyze the physicochemical structure characteristics of the blank PU foam in the reactor.

**Figure 2:**
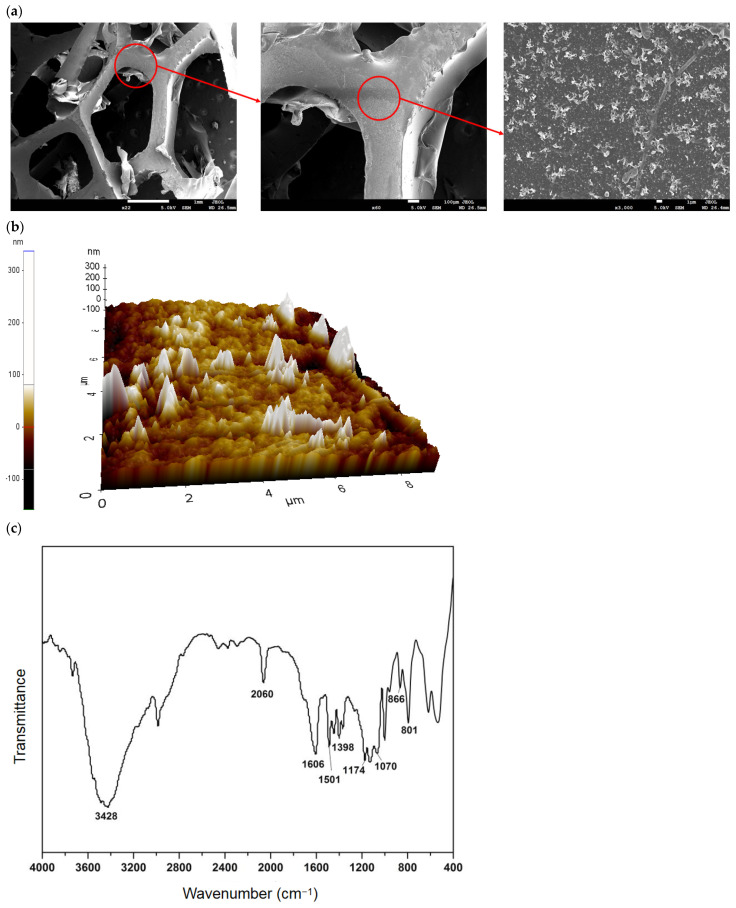
Characteristics of original bio-carrier: (**a**) SEM observation; (**b**) AFM observation; (**c**) FTIR spectrum.

The SEM in Figure 2a shows that the porous mesh structure was distinctly observed in the PU foam with a millimeter-sized hole and high porosity in terms of the filler parameters. This feature ensured the provision of a large surface area and a stable environment for the rapid formation of the biofilm developed onto the surface and interspace of the bio-carriers together. Moreover, the porous mesh structure formed by PU foams not only gives the filler a higher porosity but also enables the spitting of air bubbles carried from the substrates, hence increasing the utilization of oxygen and facilitating oxygen and mass transfer [32] from the substrate to the biofilm inside. Meanwhile, the SEM also displays that the bio-carrier has a rugged surface with unevenly distributed small spines. According to AFM in Figure 2b, the root-mean-square roughness of the PU foam was up to 41.31 nm. Such a high roughness means a favorable spatial microstructure of the bio-carrier for microbial attachment and adhesion [33].

The PU foam analyzed by FTIR in Figure 2c found the highest spectrum absorption peak at nearly 3428 cm^−1^. The broad and strong absorption peak represents a hydroxyl stretching vibration. A sharp peak with moderate intensity could be observed at 2060 cm^−1^, belonging to C≡C stretching vibration absorption peak. Moreover, it was also observed that the width of the peak at 1600 cm^−1^ was broader due to the superposition of the NH_2_-based shear vibrational absorption with the C≡C of the benzene nucleus. Additionally, a stronger NH group bending vibrational absorption peak appeared near 1400 cm^−1^. These FTIR observations suggest that these hydrophilic and cationic active groups, such as hydroxyl, amine, and imine groups, could immobilize and enrich electronegative bacteria on the surface and interface of the bio-carrier. Large specific surface area, abundant porosity, and high roughness also help to develop the biofilm and retain the biomass even at the shear force of liquid flow and airflow, thereby promoting wastewater treatment efficiency.

### 3.2. Nitrogen Removal Performance

**Figure 3:**
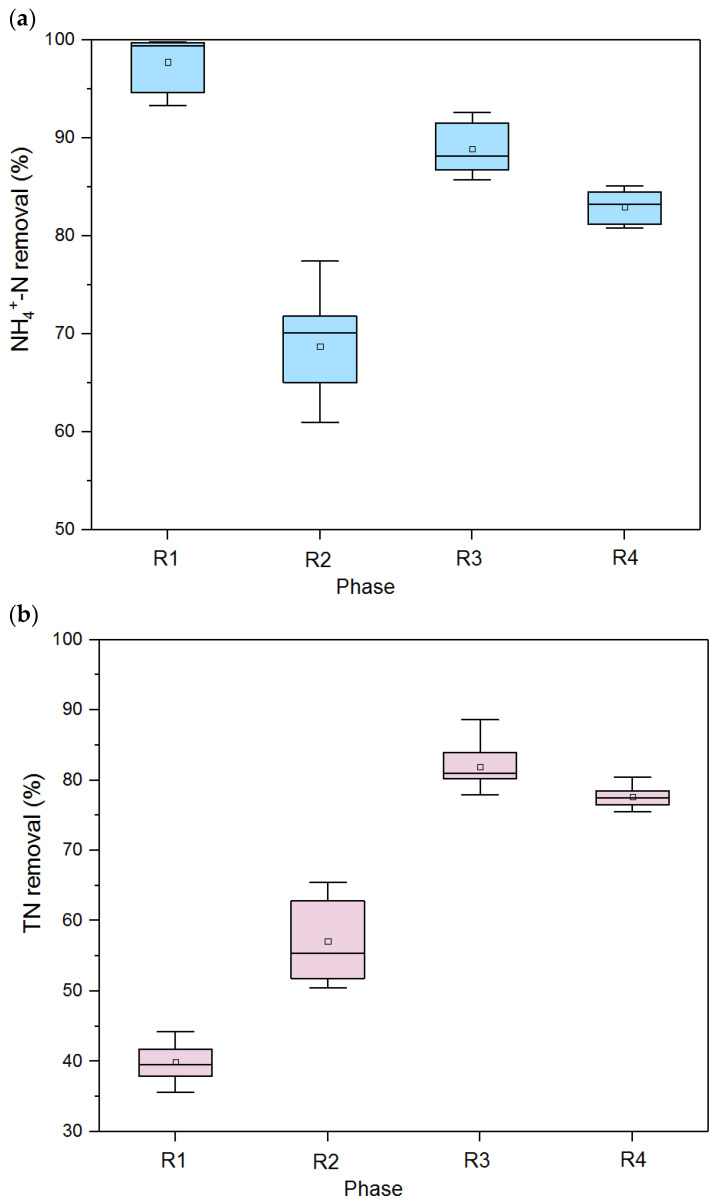
Boxplots of nitrogen removal performance: (**a**) ammonia removal efficiency; (**b**) TN removal efficiency under four scenarios (R1–R4). The box shows 25%, 50%, and 75% percentiles, and the error bar indicates 5% and 95% percentiles.

After the start-up of the biofilm reactor, the overall performance was investigated. In terms of carbon removal, the average COD removal efficiencies were 92.8%, 84.5%, 93.1%, and 92.7% for R1, R2, R3, and R4, respectively, which were always satisfactory during the whole experiment. In contrast, the nitrogen removal in SBPBBR differed sharply. Figure 3 demonstrates the boxplot of long-term operating data of nitrogen removal. In R1, excellent average ammonia removal efficiency (ARE) with 97.7% could be achieved at C/N of 2.0, CT of 24 h, and DO of 1.3 mg/L. However, nitrogen removal efficiency (NRE) was quite unsatisfactory, with an average TN removal of only 39.9%. Low-C/N and low organic loading rate reduced organic carbons for denitrification. While CT was shortened from 24 h to 12 h in R2, the NRE averagely increased by 17.8%, as more COD in the feed could be utilized by denitrifiers. On the other hand, the average ARE sharply decreased by 29% due to the doubling of organic loading. In R3, C/N was adjusted to 3.0, while other parameters were kept unchanged; as high as 88.9% ARE and 81.9% NRE on average could be simultaneously obtained in the system. It was assumed that a relatively high C/N ratio could boost the rapid development of an anoxic micro-environment and enrichment of anammox bacteria observed by the red biofilm, hence facilitating nitrogen removal capacity. However, when the DO level further increased to 2.6 mg/L in R4, declines in both ARE and NRE were found. The possible reason for that was that the increasing bulk oxygen might destroy the stability of the anammox consortium in the anoxic layers of the biofilm. Taken together, nitrogen removal could be optimized by adjusting key operating parameters such as C/N and DO in the reactor aspect. However, from a microscopic point of view, biofilm development and EPS characteristics related to the microenvironment also affected the nitrogen removal of the biofilm reactors. Therefore, further analysis of biofilm and EPS characterization combined with nitrogen removal under different operating conditions is required.

### 3.3. Morphology Analysis of Biofilm

**Figure 4:**
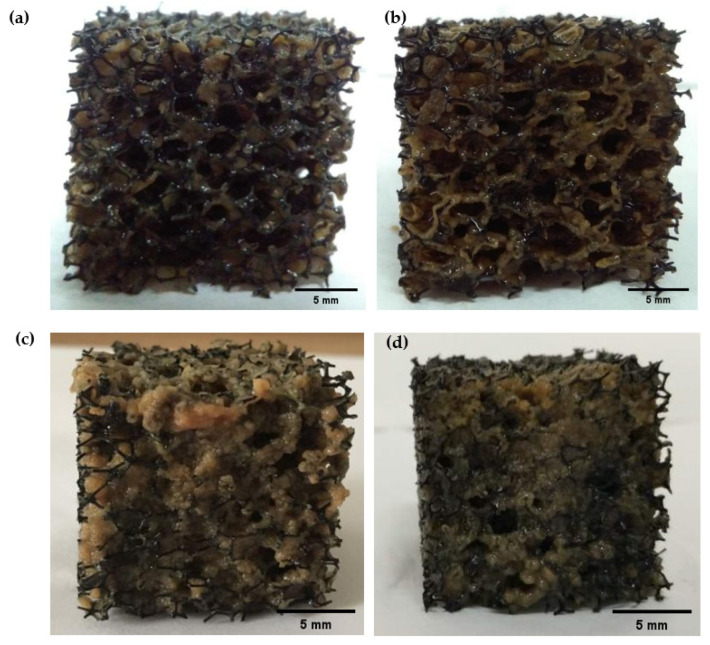
Photos of biofilms grown onto bio-carriers under four scenarios: (**a**) R1; (**b**) R2; (**c**) R3; (**d**) R4. (Scale bars: 5 mm).

**Figure 5:**
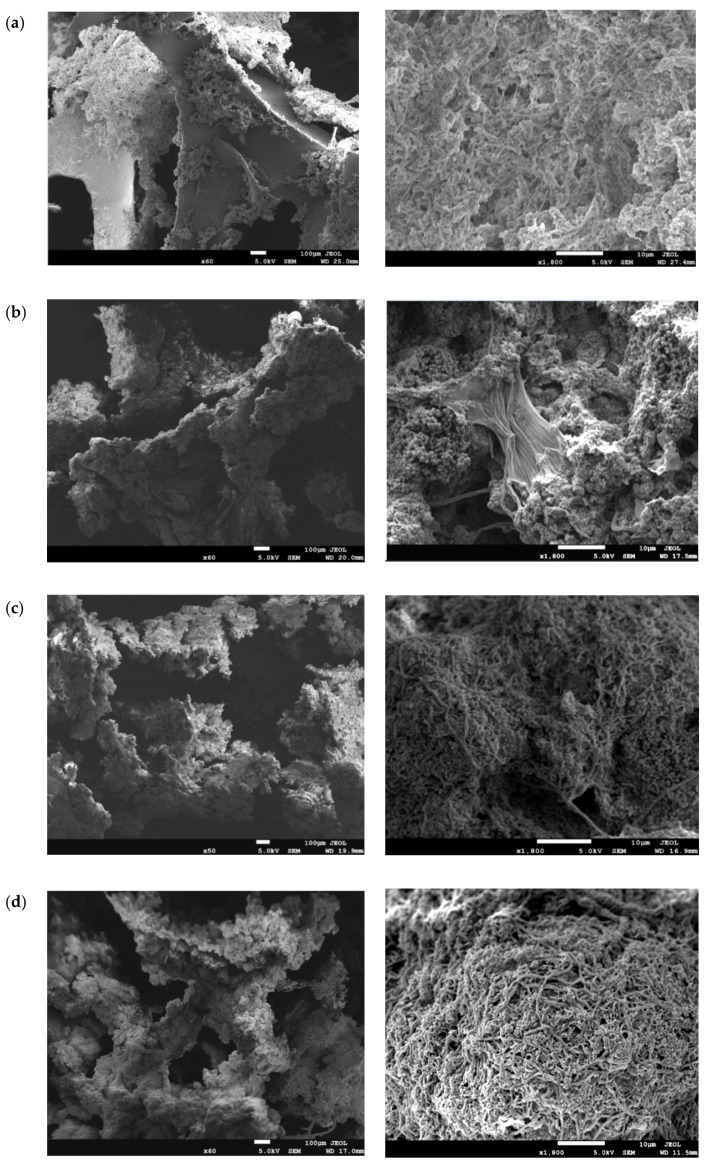
SEM observations of biofilms under four scenarios: (**a**) R1; (**b**) R2; (**c**) R3; (**d**) R4. (Scale bars: 100 μm and 10 μm).

The PU foams in different scenarios were sampled and photographed to further reveal the morphological and structural characteristics of the biofilm under different operating conditions. Figure 4 and Figure 5 display the bio-carrier morphology and biofilm SEM observation in four scenarios, respectively.

In R1, the biofilm in Figure 4a appeared light yellow but still thin with insufficient biomass (5220 mgVSS/L), although the reactor was operated for a period, indicating the weak adhesive ability of the biofilm. SEM in Figure 5a observed some tiny filamentous structures, which acted as a skeleton to entangle and link the polymers so that the biofilm could be firmly bound within the PU foam. Moreover, the pores and roughness of this structure increased the contact area between the biofilm and organic matter, reduced the destructive effect of water shear on the biofilm, and facilitated the mass transfer, thus forming a thicker and stronger biofilm. However, the biofilm was not so compact and mature in this scenario.

In R2, the biofilm in Figure 4b became yellowish brown, and the biofilm amount increased to 8035 mgVSS/L, indicating enhanced adhesiveness of the biofilm due to increased substrate loading rates in the feed. SEM observation in Figure 5b shows that the biofilm biomass increased gradually under the action of self-growth and active groups, which were on the surface of the PU foam. The filament gradually disappeared, the biofilm structure became dense, and the microbes were clearly dominated by coccus.

Interestingly in R3, it is worth noting that the biofilm turned light red in Figure 4c when the C/N was adjusted from 2 to 3. Meanwhile, the biomass attached to the bio-carrier was up to 10,256 mgVSS/L. From the SEM observation in Figure 5c, the biofilm completely covered the surface of the PU foam. The microbes bonded more closely at this time, forming a thicker and denser biofilm structure. Further observations revealed that the multiplication of organisms in the biofilm in this scenario was more extensive and diverse, constituted by spherical and filamentous bacteria. Within a certain space range, a large number of spherical microorganisms were observed, indicating that the surface and internal void of the bio-carrier provided sufficient surface area and a stable environment for the adhesion and growth of microorganisms, which enabled the bacteria to grow steadily. Meanwhile, the red color of the biomass might indicate the existence of anammox consortium [34]. Relatively high C/N and low DO provided a key basis for nitrite accumulation, and such a biofilm structure favorably formed anoxic/anaerobic micro-layers inside the biofilm [35]. Hence, efficient shortcut nitrogen removal could be achieved within an anoxic/anaerobic micro-environment inside the biofilm [36] due to the co-existence of anammox and denitrifiers, which could be proved by high-throughput sequencing based on Figure S1. These findings were consistent with the highest nitrogen removal efficiency during the experiment (Figure 3b).

In R4, the red of the biofilm became significantly weakened and even vanished, and the PU foam gradually changed to yellowish brown in Figure 4d. This is probably due to the reduction of the anoxic zone and the inactivation of anammox with increasing aeration intensity, although the biomass of 10,253 mgVSS/L in this scenario was similar to that in R3. Biological phase analysis in Figure 5d further found that spherical bacteria decreased significantly, while the microbial structure was dominated by rod-shaped bacteria. Elevation of DO level caused the decrease in anoxic layers within biofilm and deterioration of anammox species. The mentioned-above morphological results of the biofilms highly corresponded with reduced nitrogen removal in this scenario.

### 3.4. FTIR Analysis of EPSs

**Figure 6:**
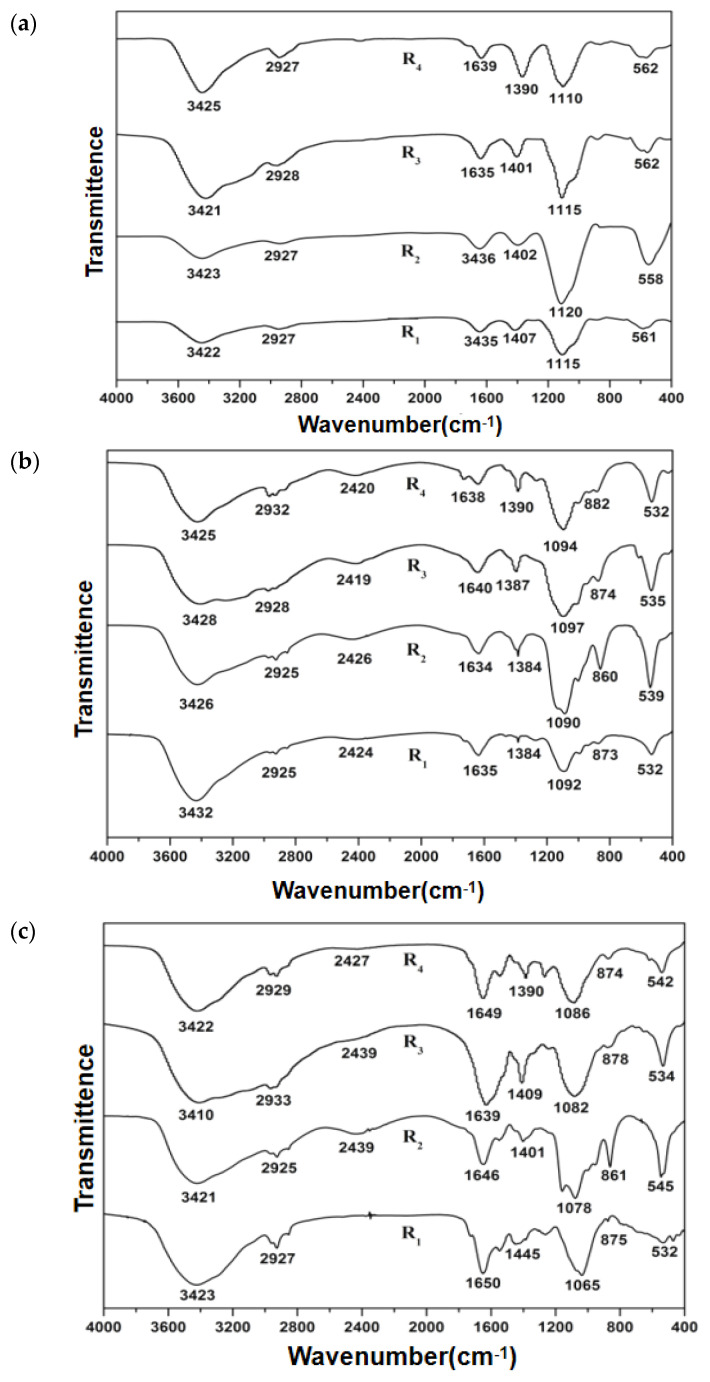
FTIR spectra of EPSs under four scenarios (R1–R4): (**a**) S-EPS; (**b**) LB-EPS; (**c**) TB-EPS.

To identify the functional groups in EPSs, FTIR spectroscopy as an effective characterization technology for the identification and structural analysis of compounds such as carbohydrates and secondary protein structures [36,37] was used in this experiment. The FTIR spectra of EPS components in different scenarios are depicted in Figure 6.

From the figure, strong and broad absorption peaks occurred within the range between 3410 cm^−1^ and 3435 cm^−1^ for EPS in all the scenarios. These were attributed to the stretching vibrations of the N-H and O-H, which could be the constituent groups of carbohydrates and amino acids in proteins of the biofilms [25]. There was another weak absorption peak at 2920 cm^−1^, indicating the anti-symmetric stretching vibration of C-H of the fatty compounds [29,38]. Moreover, the absorption peak of C=O stretching vibration appeared at 1635–1650 cm^−1^, which was lower than that of pure primary, secondary, and tertiary amide C(N)=O absorption peak (1690–1650 cm^−1^), probably due to the conjugative effect of C=C. This was considered to be the amide peak I [39], which proved that the protein was the main component of EPSs. Additionally, there was a relatively obvious absorption peak at around 1100 cm^−1^ for stretching vibration of C-O in arene, carbohydrates, or carbohydrate-like substances [38,40].

All EPS samples contained absorption peaks at 3410–3435 cm^−1^, 2920 cm^−1^, 1640 cm^−1^, and 1100 cm^−1^, indicating the presence of proteins and polysaccharides of EPS in different scenarios, which might play an important role in maintaining biofilm stability [41]. Furthermore, by comparing the EPS spectra in the same scenario, it could be found that the number of peaks formed by TB-EPSs was slightly more than that of S-EPSs and LB-EPSs from 1000 cm^−1^ to 2000 cm^−1^, demonstrating that the composition of TB-EPSs was more complex than that of S-EPSs and LB-EPSs.

Among the samples under four scenarios, although the distributions of the peaks were similar, the intensity and position of the peaks were changed, particularly in R3. In S-EPSs, the intensity of the peak at nearly 3420 cm^−1^ was the highest in R3. Compared to other scenarios, its peak had an obvious blueshift in TB-EPSs. While at nearly 2920 cm^−1^ and 1650 cm^−1^, slight redshifts in these peaks were observed in R3. Moreover, the intensity of the secondary peak at nearly 1400 cm^−1^ representing the C=O symmetrical stretching from the amino acid [42] increased sharply. These FTIR results clearly revealed the changes in the molecular structure of key functional groups constituting EPSs, which could provide a useful signal for nitrogen removal performance.

### 3.5. 3D-EEM Analysis of EPSs

**Figure 7:**
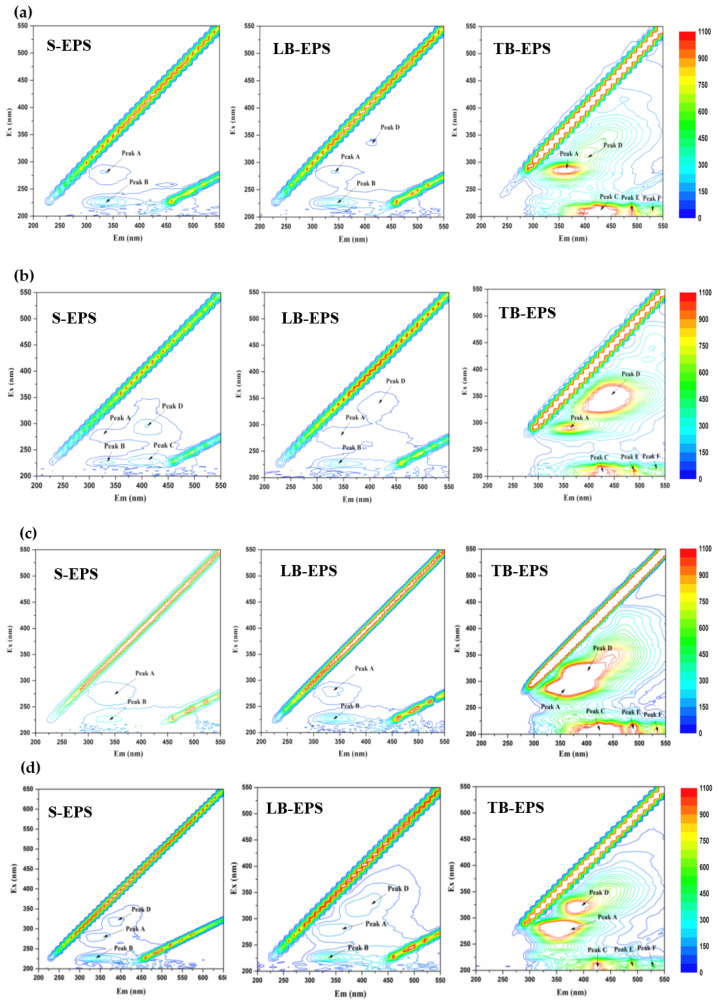
3D-EEM spectra of EPSs under four scenarios: (**a**) R1; (**b**) R2; (**c**) R3; (**d**) R4.

In biofilm EPSs, the substances usually contain fluorescent groups, which can be used to analyze the changes in the composition, content, and type of EPS during the biological reaction process [28]. Figure 7 demonstrates the 3D-EEM fluorescence spectra of TB-EPSs, LB-EPSs, and S-EPSs in different scenarios.

There was a total of six peaks contained in the EPSs. Peak A located at Ex (275–280) nm and Em (325–345) nm, and peak B was located at Ex (220–250) nm and Em (325–350) nm. Both peaks were regarded as protein-like peaks associated with aromatic protein-like substances such as tyrosine (Peak A) and tryptophan protein-like substances (Peak B) [43]. The peak D is located at Ex (300~350) nm/Em (390~425) nm, belonging to the fluorescence response region of polycarboxylate humic acid-like substances [44]. Moreover, the peaks C, E, and F were at Ex/Em = (200–220) nm/(385–465) nm, Ex/Em = (200–220) nm/(485–495) nm, and Ex/Em = (200–220) nm/(530–540) nm, respectively, indicating the existence of fulvic acid-like substances [45].

According to Figure 7, TB-EPSs contained more EPSs than S-EPSs and LB-EPSs in the same scenarios, as well as more abundant proteins and byproducts secreted by microorganisms, indicating that TB-EPSs played a more important role in protecting the microbial community in the biofilm. This was consistent with the result of FTIR. Furthermore, as an important part of biofilms, the variations in fluorescence peaks and fluorescence intensities detected by EPSs on the 3D-EEM fluorescence spectra indicate the strong response ability of extracellular biofilm polymers to the environmental changes that arise from operating conditions.

As seen in Figure 7, tryptophan protein-like substances and aromatic protein-like substances were the two main substances of S-EPSs. In Figure 7b, peak C suddenly appeared at the shoulder of peak B in R2, indicating that S-EPSs contained fulvic acid-like substances. Peak D, representing the fluorescence response region of humic acid-like substances, appeared simultaneously with peak C but disappeared in Figure 7c in R3.

TB-EPSs contained five peaks A, C, D, E, and F. Peak A had a stable fluorescence intensity in R1-R2, which responded to the fluorescence region of tryptophan proteins. Particularly, polycarboxylic humic acids (peak D) were enriched in large quantities, a crucial component of EPSs, formed by the adsorption of humic acids in wastewater and promoting the activity of esterase [46,47]. While in R3, the fluorescence intensity of peak A surged, while that of peak D weakened and connected with peak A to form a whole. Moreover, the fluorescence intensity of peak F, representing fulvic acid-like substances, also increased. In R4, the fluorescence intensity of peaks A, D, and F decreased simultaneously, leading to the separation of peaks A and D. Based on these fluorescence profiles, it was speculated that the production of tryptophan protein-like substances and humic acid-like substances was responsible for promoting TN removal. Sufficient tryptophan protein-like substances were beneficial to maintain the stability of the biofilm structure, thus promoting the synergistic growth of a variety of nitrogen-removal functional bacteria in the biofilm. Moreover, humic acid-like and fulvic acid-like substances produced by microbial metabolisms could also be used as alternative carbon sources for denitrification, ensuring improved nitrogen removal at a low C/N ratio in the SBPBBR.

Table S2 summarizes the position and fluorescence intensity of EPS peaks based on 3D-EEM analysis. Compared with S-EPSs and LB-EPSs, TB-EPSs containing a lot of protein substances and microbial by-products were more closely bound to the surface of biofilm cells and were easily biodegraded, enabling some microorganisms in biofilm to potentially turn to TB-EPSs as an energy source in the case of insufficient carbon source, leading to the degradation of large molecules and the generation and alteration of small functional groups in TB-EPSs [48]. Moreover, the total fluorescence intensity of TB-EPSs was the highest in R3, according to Table S2. This result fitted perfectly with its highest TN removal. It was very likely that more diversified protein-like substances secreted by EPSs could improve carbon metabolisms and produce sufficient endogenous carbon sources [49]. Accordingly, biofilm robustness and nitrogen removal-associated bacteria enrichment were enhanced under the optimal operating mode.

In addition, somewhat of position shifts in fluorescence peaks of EPSs could be observed shown in Table S2. Redshift may occur in different scenarios, indicating that the position of the fluorescence peak shifts to a long-wave region due to an increase in the number of some functional groups, such as carbonyl, hydroxyl, amino, and carboxyl [45]. In TB-EPS, the Ex/Em at peak A in R2 and R3 redshifted by 6/0 nm and 3/5 nm, respectively, compared with peak A in R1. On the other hand, some peaks had a blueshift to the short-waves region due to the decrease in some functional groups, including aromatic hydrocarbons and conjugated bond chains [50]. The Ex/Em of peak A in R4 blueshifted by 6/11 nm compared to R1. The occurrence of redshift and blueshift in TB-EPSs implied that the composition and chemical structure of EPSs were changed in accordance with the results of FTIR analysis. Unlike TB-EPSs, the positions of the fluorescence peaks of S-EPSs and LB-EPSs varied little for different scenarios. Therefore, these evident transformations in the fluorescence spectra of EPSs could act as effective indicators for the nitrogen removal performance of SBPBBR.

## 4. Conclusions

In this study, the essential role of EPSs was specifically deciphered in enhancing nitrogen removal from high-ammonia and low-C/N wastewater in an SBPBBR system. Firstly, SEM, AFM, and FTIR characterizations of the PU foam indicated that the attachment and microbial adhesion of the biofilm were determined by its special physicochemical and structural properties. These bio-carrier properties provide a key basis for optimizing the reactor to achieve satisfactory nitrogen removal performance. Additionally, the visible morphology and microbial phase of the biofilm were major factors in determining nitrogen removal. FTIR and 3D-EEM spectroscopy analysis showed that the nitrogen removal was relative to the composition, structure, content, and variety of EPSs. Moreover, significant shifts in the number, intensity, and position of EPS fluorescence peaks of TB-EPSs might be responsible for influencing nitrogen removal in biofilm reactors.

## Figures and Tables

**Table 1:** Composition and trace element of synthetic wastewater.

Main Elements	mg/L
Glucose	600; 900
NH_4_Cl	300
KH_2_PO_4_	60
NaHCO_3_	1700
Trace element	1 mL/L
**Trace element solution contained**	
EDTA	5000
CoCl_2_·6H_2_O	1500
MnSO_4_	120
H_3_BO_4_	150
NiCl_2_·6H_2_O	190
ZnSO_4_·7H_2_O	120
FeCl_3_	1500
CuSO_4_	30

**Table 2:** Operational conditions of the SBPBBR under four scenarios.

Scenario	COD mg/L	NH_4_^+^-N mg/L	C/N -	Time/Cycle h	DO mg/L	Number of Cycles
R1	600	300	2	24	1.3	26
R2	600	300	2	12	1.3	34
R3	900	300	3	12	1.3	32
R4	900	300	3	12	2.5	20

## Data Availability

The data presented in this study are available on request from the corresponding author.

## Supplementary Materials

The following supporting information can be downloaded at: https://www.mdpi.com/article/10.3390/polym15061510/s1, Figure S1: Microbial community at genus level (in R3) based on high-throughput sequencing analysis; Table S1: Parameters of the PU foam; Table S2: Main fluorescence parameters of 3D-EEM under four scenarios (R1–R4).
