# Supplementary material for: Decoding the Role of Extracellular Polymeric Substances in Enhancing Nitrogen Removal from High-Ammonia and Low-C/N Wastewater in a Sequencing Batch Packed-Bed Biofilm Reactor
Source: Polymers (Basel). 2023 Mar 17;15(6):1510. doi: 10.3390/polym15061510 (PMC10051956; doi:10.3390/polym15061510)
Supplement: Supplementary file 1 [file polymers-15-01510-s001.zip › polymers-2190951-supplementary.pdf]

Supplementary Materials

Table S1. Parameters of the PU foam.

Item	Unit	Value
Size	cm × cm × cm	2.5×2.5×2.5
Specific surface area	m^2/g	≥23.3
Weight	kg/m^3	23±0.5
Density	kg/m^3	approximately 1000
Porosity	%	≥95
Stacking density	n/m^3	8000

Table S2. Main fluorescence parameters of 3D-EEM under four scenarios (R1-R4).

EPS	Scenario	Peak A		Peak B		Peak C		Peak D		Peak E		Peak F	
		Ex/Em	FI*	Ex/Em	FI	Ex/Em	FI	Ex/Em	FI	Ex/Em	FI	Ex/Em	FI
S-EPS	R1	280/340	102	226/330	282	-	-	-	-	-	-	-	-
	R2	284/320	76	226/330	167	230/410	198	294/410	156	-	-	-	-
	R3	274/330	113	226/330	292	-	-	-	-	-	-	-	-
	R4	280/330	133	226/340	333	-	-	320/380	118	-	-	-	-
LB-EPS	R1	282/340	106	226/340	294	-	-	336/410	56	-	-	-	-
	R2	282/330	95	226/330	231	-	-	336/410	72	-	-	-	-
	R3	280/340	128	228/345	297	-	-	-	-	-	-	-	-
	R4	280/330	133	226/340	333	-	-	320/380	118	-	-	-	-
TB-EPS	R1	284/365	1014	-	-	210/425	1014	310/400	611	208/490	1014	208/530	770
	R2	290/365	995	-	-	208/425	1016	346/440	1016	210/490	1016	212/530	609
	R3	287/370	1012	-	-	210/425	1012	317/395	1012	210/490	1012	208/530	1012
	R4	278/354	974	-	-	208/425	974	320/385	974	212/490	861	208/530	524

* Fluorescence intensity of the peak.

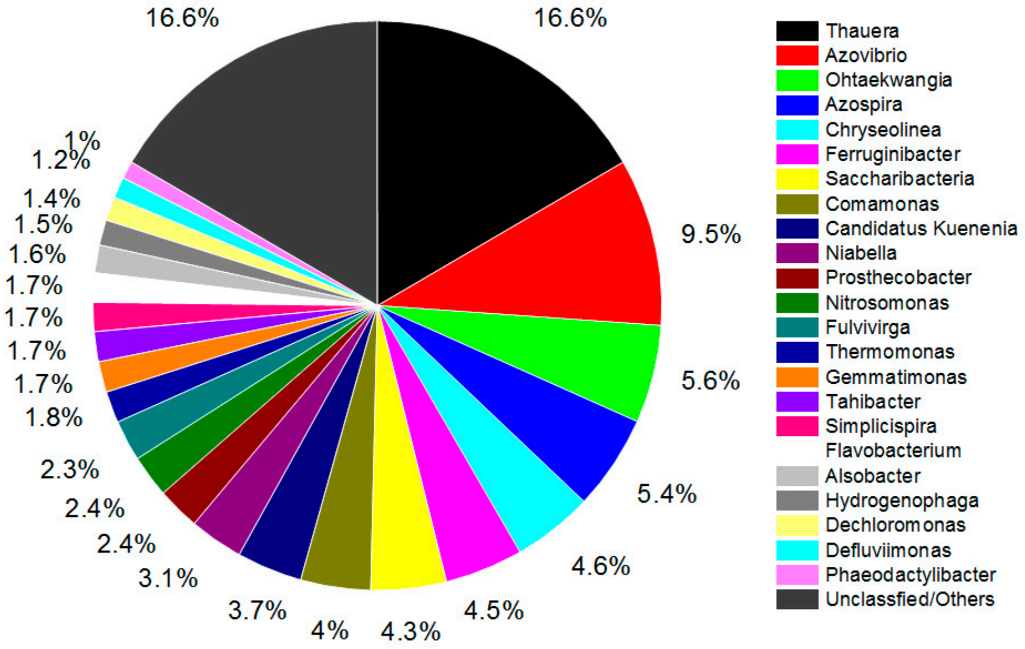

Figure S1. Microbial community at genus level (in R3) based on high-throughput sequencing analysis
